# X-linked Alport syndrome presenting in mother and son with the same unique histopathological features

**DOI:** 10.1007/s40620-024-01942-7

**Published:** 2024-04-26

**Authors:** Nicolas A. D. Bergeron, Alexandre P. Garneau, Mathieu Rousseau-Gagnon, Julie Riopel, Paul Isenring

**Affiliations:** 1 Service of Nephrology, L’Hôtel-Dieu de Québec Research Center, CHU de Québec-Université Laval, 10 McMahon Street (Room 3852), Québec, QC G1R 2J6 Canada; 2 Service of Pathology, CHU de Québec-Université Laval, Québec, QC G1R 2J6 Canada; 3 Service de Néphrologie-Transplantation Rénale Adultes, Hôpital Necker-Enfants Malades, AP-HP, Inserm U1151, Université Paris Cité, Rue de Sèvres, Paris, France

**Keywords:** Alport syndrome, IgA nephropathy, *COL4A5*, Collagen assembly, Bowman’s capsule

## Abstract

Alport syndrome has been linked to three different genes, that is, *COL4A3*, *COL4A4* and *COL4A5*. It is characterized by progressive and non-specific glomerulosclerosis with irregular thickening of the glomerular basement membrane (GBM). At times, the histopathologic picture is dominated by lesions that are consistent with focal and segmental glomerulosclerosis or IgA nephropathy. Here, we report the cases of two related individuals (mother and son) who were diagnosed with *COL4A5*-related Alport syndrome due to a missense variant (p.Gly1170Ser) in a G-X-Y repeat and found to present the same highly unusual histopathological abnormalities on their kidney biopsies. One of the abnormalities shared, which does not appear to have been reported, was reduced COL4A5 immunolabeling that was limited to Bowman’s capsule even though the ultrastructure of the GBM was distorted. The other abnormality was superimposed segmental IgA deposition in both individuals, accompanied by mesangial changes in the mother. We feel that these findings provide novel insight into the mechanisms of disease manifestation in Alport syndrome. They suggest, in particular, that collagen expression and/or assemblies in Bowman’s capsule is more vulnerable to missense mutations in *COL4A5* than elsewhere in the kidney. Our findings also suggest that certain coinherited gene polymorphisms act as unexpectedly important phenotypic determinants in *COL4A*-related disorders.

## Introduction

Alport syndrome occurs at a prevalence of ~ 1% through mutations in *COL4A3* (2q36.3), *COL4A4* (2q36.3) or *COL4A5* (Xq22.3) [1]. Progressive kidney disease is the main manifestation of this syndrome and is generally accompanied by pathognomonic distortions of the glomerular basement membrane (GBM) with varying degrees of glomerulosclerosis and tubulointerstitial fibrosis [2]. At times, it can develop without such distortions and be associated with IgA nephropathy (IgAN) or an IgA-like nephropathy [3, 4].

There are three types of collagen IV assemblies in the kidney: the COL4A3-4-5 and COL4A1-1-2 assemblies, which are found in the GBM and Bowman’s capsule, and the COL4A5-5-6 assembly, which is found in Bowman’s capsule alone [1]. In many cases, a pathogenic mutation in one of these genes hinders trimerization and/or expression of all protomers within an assembly [1]. When the mutation affects *COL4A5*, the gene product is often absent from both the GBM and Bowman’s capsule, and when the mutation affects *COL4A3* or *COL4A4*, COL4A5 is often absent from the GBM but always present in Bowman’s capsule [1].

Here, we report a unique form of *COL4A5*-related Alport syndrome that we have uncovered in two individuals from the same family. To us, the observed presentation was of exceptional interest for at least two reasons. First, it provided valuable insight into the effect of the mutation on the assembly of the COL4A5-based trimers. Second, it also suggested that both the molecular as well as clinical repercussions of the primary gene defect were shaped by a coinherited predisposition.

## Presentation of cases

A 48-year-old woman known for systemic hypertension was referred to our Nephrology Service for longstanding microscopic hematuria and progressive chronic kidney disease (CKD) with an estimated glomerular filtration rate (eGFR) of 40 mL/min/1.73 m^2^ and proteinuria of 416 mg/day at presentation. At the initial visit, the patient had two sons in apparent good health and reported no family history of kidney disease or deafness.

A renal biopsy ordered to clarify the cause of CKD was suggestive of chronic IgAN (results not shown). In particular, light microscopy showed diffuse mesangial expansion with focal areas of hypercellularity, moderate glomerulosclerosis and chronic tubulointerstitial disease, immunofluorescence showed segmental IgA deposition in the mesangium and capillary loops, and electron microscopy showed occasional mesangial and intramembranous electron-dense deposits. COL4A2 and COL4A5 distribution in the GBM and Bowman’s capsule was otherwise uniform, and GBM thickness normal (227–318 nm).

**Fig. 1:**
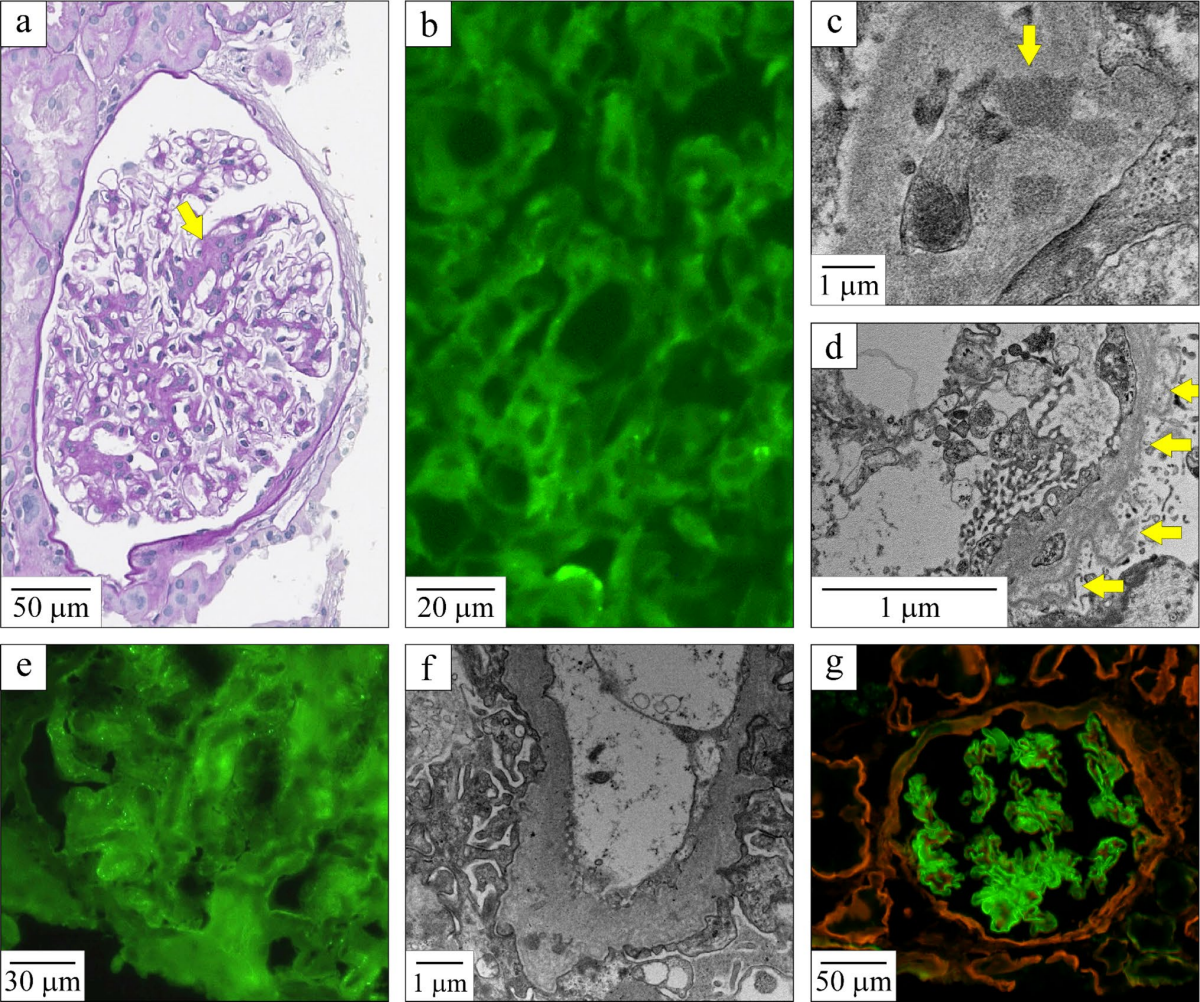
Results of the kidney biopsies. Images in the upper row are from the second biopsy in the mother and images in the lower row from the biopsy in the son. **a** Light microscopy (PAS). Glomerulus showing mesangial expansion with segmental mesangial hypercellularity (arrow). **b** Immunofluorescence. Mild segmental IgA deposition. **c** Electron microscopy. Paramesangial and mesangial electron-dense deposits (arrow). **d** Electron microscopy. GBM showing splitting of the lamina densa with multilamellation giving rise to a basket-weave appearance (arrows). Thickness ranged from 250 to 404 nm (normal in females: 215–395 nm). **e** Immunofluorescence. Weak segmental IgA deposition. **f** Electron microscopy. GBM showing splitting of the lamina densa with multilamellation giving rise to a basket-weave appearance. **g** Immunofluorescence. COL4A5 immunolabeling (in green) is continuous and linear in GBM but absent in Bowman’s capsule and proximal tubular basement membranes (marked in red through COL4A2 immunolabeling). The figure was prepared with the CorelDRAW^®^ Graphics Suite software

A second biopsy carried out 18 months later for worsening of the kidney function revealed similar findings by light microscopy, immunofluorescence and electron microscopy (see Fig. 1a–c, respectively). However, two additional abnormalities were noted: (1) immunolabeling for COL4A5 in Bowman’s capsule appeared paler as well as discontinuous (not shown) and (2) the GBM exhibited varying degrees of thickness (250–404 nm) with segmental remodeling, multilamellation and intramembranous granular particles (Fig. 1d).

Five years later, one of the patient’s sons was found to have proteinuria (729 mg/day) and hematuria with an eGFR of more than 120 mL/min/1.73 m^2^. He was 29 years old and otherwise in good health. A kidney biopsy showed mild global glomerulosclerosis by light microscopy along with chronic tubulointerstitial disease (not shown), weak segmental IgA deposition by immunofluorescence (Fig. 1e) and GBM alterations by electron microscopy with areas of thickening or thinning, multilamellation and intramembranous granular particles (Fig. 1f). Immunolabeling for COL4A5 in Bowman’s capsule was also absent but preserved in the GBM, and immunolabeling for COL4A2 was normal (Fig. 1g).

Eventually, next generation exome sequencing revealed that both mother and son carried the pathogenic p.Gly1170Ser (c.3508G>A) missense mutation in exon 39 of *COL4A5*. This variant was initially detected in the son through a 343-gene panel and later confirmed in the mother by site-specific testing. It affects the first G of a G-X-Y repeat within the distal third of the collagenous domain and has been associated with Alport syndrome in at least 5 non-related families. No other variants of interest could be identified in the 51 exons of *COL4A5* or among the genes tested.

## Discussion

These two cases of *COL4A5*-related nephropathy were remarkable for various reasons. For instance, we found that immunolabeling for COL4A5 could be decreased or lost in Bowman’s capsule while preserved in the GBM, a finding that has never been reported in this condition before. Additionally, the ultrastructural abnormalities seen in both cases were consistent with Alport syndrome but also came with variable degrees of IgA deposition. Lastly, the p.Gly1170Ser mutation was associated with a unique yet similar histopathological portrait in both mother and son.

It is intriguing that in contrast to Bowman’s capsule, the GBM showed preserved expression of COL4A5 in the two family members and did so even if it was ultrastructurally abnormal. One explanation could be that the COL4A3-4-5 trimers were more efficiently processed than the COL4A5-5-6 trimers by harboring only one mutation per trimer instead of two. This explanation would be in keeping with the observation that G-changing missense mutations in several of the COL4A5 G-X-Y repeats have been associated with more favorable outcomes than truncating mutations but still found to alter the structure of the GBM [5, 6].

As mentioned, Bowman’s capsule in the mother showed a segmental decrease in COL4A5 expression on the second renal biopsy. This other finding was not unexpected in that the gene defect affected chromosome X and was thus bound to be inactivated in only a subgroup of cells within the kidney. It could also be for the same reason that COL4A5 expression in Bowman’s capsule appeared normal in the mother on the first biopsy, i.e., that it was preserved because the glomeruli sampled were from a region where X-inactivation was favorable.

In view of the gene defect uncovered, the other interesting aspect of our report was the presence of IgA deposition in both cases and mesangiopathy in the mother. While a recent exome-wide analysis of familial IgAN from 46 pedigrees has already led to the identification of gene defects in *COL4A5* [7], it has only been seldom reported afterwards that the two conditions could coexist. Our findings should thus be seen as a timely contribution to this emerging context. They also suggest that IgAN in Alport syndrome can take a while to manifest in its typical form as it was indeed more manifest in the mother.

As for the reason that could explain why a gene defect in *COL4A5* can lead to IgAN, some authors have argued that GBM thinning could simply allow IgA to translocate more readily from blood to mesangium and elicit a pathological response at this new location [3]. Such a scenario would be congruent with the observation that IgAN has also been described with gene defects in *COL4A3* and *COL4A4*. However, one would then wonder why mesangial deposition of the other immunoglobulins or of C3 is not a very common finding in Alport syndrome.

In our view, a more plausible explanation would be that the susceptibility to develop a COL4A3-, COL4A4- or COL4A5-related form of IgAN is driven by coinherited gene polymorphisms. For instance, this renal lesion was seen in our blood-related cases while it has not previously been associated with the COL4A5 p.Gly1170Ser mutation. Along the same line, many individuals who were found to carry pathogenic mutations in *COL4A3*, *COL4A4* or *COL4A5* were also blood-related [7].

As it stands, there is evidence to suggest the involvement of IgAN-associated polymorphisms in Alport syndrome. In particular, a genome-wide association study of sporadic IgAN has led to the identification of a susceptibility locus (2q36) that encompasses the *COL4A3* and *COL4A4* genes [8] and that could thus harbor a polygene through which IgA1 processing is coordinated. In the case of *COL4A5*, *VSIG1* could correspond to another polygene of interest given that it is involved in gastrointestinal immune regulation and is also localized in locus Xq22.3 [9].

A question that will need to be addressed in the course of future investigations is whether COL4-related IgAN could be treated as idiopathic IgAN, especially if galactose-deficient IgA1 were to be involved in disease progression. This question will become particularly relevant if complement pathway inhibitors or B-cell-directed strategies in idiopathic IgA were eventually found to be more effective and better tolerated than the previously described approaches.

In conclusion, we have reported for the first time two cases of X-linked Alport syndrome where the only structure affected by a decrease in COL4A5 immunolabeling was Bowman’s capsule and where neither *COL4A3* nor *COL4A4* could have thus been at cause. The cases described were also striking in being blood-related and both showing glomerular IgA deposition. These observations suggest that the molecular and clinical impacts of the mutation identified were conditioned in mother and son by a coinherited gene defect in or near the Xq22.3 locus.

## Data Availability

Original data will be made available upon request.

## References

[1] Hudson BG, Tryggvason K, Sundaramoorthy M, Neilson EG (2003). Alport's syndrome, Goodpasture's syndrome, and type IV collagen. N Engl J Med.

[2] Nozu K, Nakanishi K, Abe Y (2019). A review of clinical characteristics and genetic backgrounds in Alport syndrome. Clin Exp Nephrol.

[3] Savige J, Harraka P (2021). Pathogenic variants in the genes affected in Alport syndrome (COL4A3-COL4A5) and their association with other kidney conditions: a review. Am J Kidney Dis.

[4] Gast C, Pengelly RJ, Lyon M (2016). Collagen (COL4A) mutations are the most frequent mutations underlying adult focal segmental glomerulosclerosis. Nephrol Dial Transplant.

[5] Hashimura Y, Nozu K, Kaito H (2014). Milder clinical aspects of X-linked Alport syndrome in men positive for the collagen IV α5 chain. Kidney Int.

[6] Gross O, Netzer KO, Lambrecht R, Seibold S, Weber M (2002). Meta-analysis of genotype-phenotype correlation in X-linked Alport syndrome: impact on clinical counselling. Nephrol Dial Transplant.

[7] Li Y, Groopman EE, D'Agati V (2020). Type IV collagen mutations in familial IgA nephropathy. Kidney Int Rep.

[8] Paterson AD, Liu XQ, Wang K (2007). Genome-wide linkage scan of a large family with IgA nephropathy localizes a novel susceptibility locus to chromosome 2q36. J Am Soc Nephrol.

[9] Zhou X, Khan S, Huang D, Li L (2022). V-Set and immunoglobulin domain containing (VSIG) proteins as emerging immune checkpoint targets for cancer immunotherapy. Front Immunol.

